# College and Hospital Notes

**Published:** 1911-11

**Authors:** 


### COLLEGE AND HOSPITAL NOTES

The 66th annual session of the University of Buffalo, Medical Department, opened on Monday, September 25. Following the time-honored custom, the regular work of the year was introduced by a lecture upon some general topic, which was delivered on this occasion by Dr. Edward R. McGuire. Dr. McGuire struck into a somewhat original vein in reading a biographic sketch of John Hunter. His treatment of the subject was very happy showing how much there was in the life in this great man to stimulate his successors to industry and exact observation.

The Medical Department has a larger freshman class than it has had for several years; more than eighty students although the entrance requirements are more severe than they have ever been before, as inorganic chemistry is one of the subjects required for entrance. In this respect, the Buffalo school is in advance of the requirements of the New York State Board of Regents.

